# Cohen Syndrome With Complex Medical Complications: A Case Report

**DOI:** 10.7759/cureus.66033

**Published:** 2024-08-02

**Authors:** Fathi S Milhem, Ameer Awashra, Husam Hamshary, Zaid Sawaftah, Amr Khaled, Noor Nabresi, Israa Salman

**Affiliations:** 1 Faculty of Medicine and Health Sciences, An-Najah National University, Nablus, PSE; 2 Department of Medicine, An-Najah National University, Nablus, PSE; 3 Pediatrics and Neonatology, Tulkarim Governmental Hospital, Tulkarm, PSE; 4 Pediatrics and Neonatology, Tulkarim Governmental Hospital, Tulkarim, PSE

**Keywords:** gastrointestinal bleeding, vps13b gene, ulcerative colitis, thalassemia major, cohen syndrome

## Abstract

Cohen syndrome (CS) is a rare autosomal recessive disorder marked by developmental delays, distinct facial features, and a variety of systemic manifestations. We present a case of a 28-year-old male previously misdiagnosed with Prader-Willi syndrome who exhibited recurrent generalized weakness, fever, fatigue, and significant hemoglobin drops requiring multiple blood transfusions due to thalassemia major. The patient displayed characteristic CS features, including developmental delays, distinct facial characteristics, morbid obesity, and heterochromia iridis. Severe gastrointestinal bleeding led to a diagnosis of ulcerative colitis (UC) via colonoscopy. Management included blood transfusions, hydrocortisone, mesalamine, and azathioprine, resulting in stabilized UC and improved overall health. CS presents with a spectrum of clinical features that overlap with other syndromic conditions, necessitating careful differential diagnosis. Early diagnosis and supportive care significantly improve quality of life and help manage complications effectively.

## Introduction

Cohen syndrome (CS), an autosomal recessive syndrome first reported in 1973 by Cohen et al., is characterized by developmental delay, distinctive facial features, and a range of systemic manifestations. These include ocular issues such as constricted visual fields and night blindness, hematologic abnormalities like leukopenia, musculoskeletal problems such as ligamentous laxity and hypermobility, and neurological signs such as brisk tendon reflexes and muscular hypotonia. Gastrointestinal challenges, cardiovascular complications, including decreased left ventricular function and valvular defects, and dental issues like prominent upper incisors and alveolar bone loss are also common. CS is primarily caused by mutations in the vacuolar protein sorting 13 homolog B (VPS13B) gene, which encodes a protein involved in intracellular transport and membrane trafficking. These mutations disrupt cellular processes, leading to the characteristic features of CS [1-3].

In this study, we present a patient with typical characteristics of CS and many complex medical complications and surgical interventions. Additionally, the patient complained of lower gastrointestinal bleeding with recurrent episodes of anemia despite receiving recurrent blood transfusions. The purpose of this report is to draw attention to such uncommon complications of CS.

## Case presentation

A 28-year-old male patient with CS, previously misdiagnosed with Prader-Willi syndrome, presented with a complex medical history, including thalassemia major and hypothyroidism. He arrived at our hospital experiencing recurrent episodes of generalized weakness, fever, fatigue, and a significant drop in hemoglobin levels, necessitating multiple hospitalizations and blood transfusions due to his thalassemia major. During this visit, he reported lower quadrant abdominal pain and a change in stool color.

**Figure 1:**
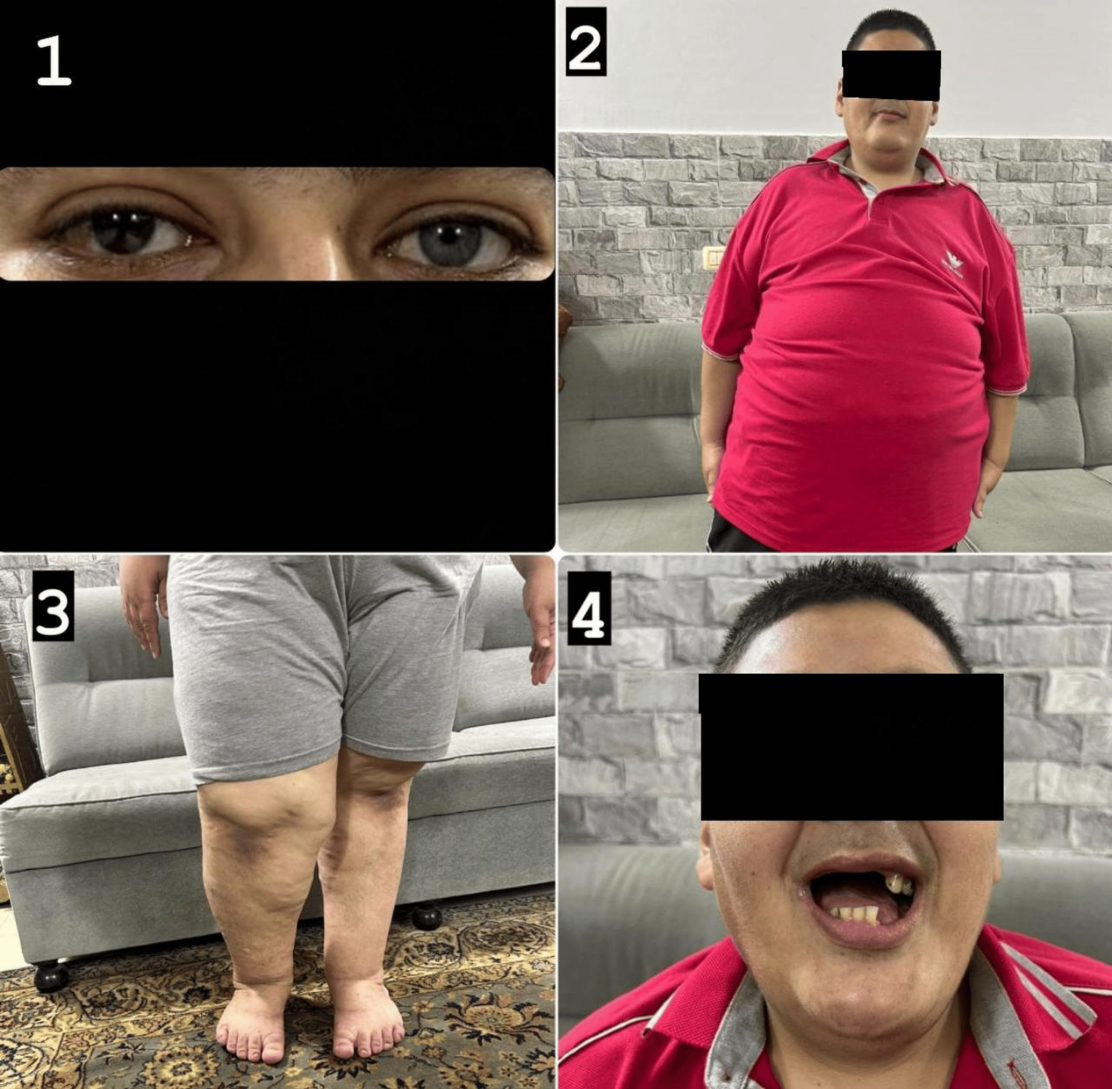
Illustrates several notable clinical findings. Image 1 reveals heterochromia iridis, characterized by different iris colors in the same individual. Image 2 depicts morbid central obesity, a severe form of obesity with significant health implications. Image 3 demonstrates anisomelia, indicating a discrepancy in leg length. Finally, Image 4 showcases dental eruptions.

In the ER, thorough investigations were conducted, including laboratory tests that revealed a hemoglobin level of 8 g/dL (Table 1), warranting admission for a blood transfusion. Upon admission, the physical examination revealed features consistent with CS, such as developmental delays in motor skills and speech, intellectual disability, distinct facial characteristics like a prominent nose, dental eruptions, heterochromia iridis, and anisomelia (leg length discrepancy) (Figure 1). Additionally, he presented with morbid obesity and tenderness in the lower quadrant of the abdomen, without anal fissures or hemorrhoids, and no active rectal bleeding at the time of assessment. However, two hours later, he began to exhibit symptoms of severe gastrointestinal bleeding, presenting with recurrent episodes of blood per rectum. It is worth noting that genetic testing confirmed the diagnosis of Cohen syndrome, identifying mutations in the VPS13B gene.

**Table 1:** Illustrates comparative laboratory values on admission day and discharge day. Hb: hemoglobin, Hct: hematocrit, RBC: red blood cell, MCV: mean corpuscular volume, WBC: white blood cell, TIBC: total iron binding capacity, CRP: C-reactive protein, ESR: erythrocyte sedimentation rate, ALT: alanine aminotransferase, AST: aspartate aminotransferase, ALP: alkaline phosphatase, BUN: blood urea nitrogen, TSH: thyroid-stimulating hormone, FT4: free thyroxine, PT: prothrombin time, PTT: partial thromboplastin time, INR: international normalized ratio.

	Hb	Hct	RBC count	MCV	WBC	Platelet count	Serum ferritin	Serum iron	TIBC	Transferrin saturation	CRP	ESR	ALT	AST	ALP	Bilirubin	BUN	Creatinine	TSH	FT4	PT	PTT	INR	Occult blood test	Fecal calprotectin
Admission day	8	24	3	74	12,000	420,000	1000	180	200	80	20	50	70	65	153	2	25	1.2	6	0.6	13	35	1.2	Positive	207
Discharge day	11	33	4.62	86	8000	362,000	577	120	377	64	7	18	40	35	102	1	15	0.9	3	1.2	12	30	1	Negative	43
Normal range	13.8–17.2	40.7–50.3	4.7–6.1	80–100	4500–11,000	150,000–450,000	30–400	60–170	240–450	20–50	<10	<20	7–50	10–40	44–147	0.1–1.2	7–20	0.6 - 1.2	0.4–4.0	0.8–1.5	11–24	25–35	0.9–1.1	Negative	<50
Unit	g/dL	%	million cells/µL	fL	cells/µL	cells/µL	ng/mL	µg/dL	µg/dL	%	mg/L	mm/hr	U/L	U/L	U/L	mg/dL	mg/dL	mg/dL	µIU/mL	ng/dL	seconds	seconds	-	-	µg/g

Further investigations, including a colonoscopy and biopsy due to suspected colonic involvement, revealed mucosal inflammation in the colon, confirming the diagnosis of ulcerative colitis (UC). The patient's management included regular blood transfusions, the administration of intravenous fluids and medications such as hydrocortisone, and close monitoring of vital signs and hemoglobin levels.

Following initial treatment, the patient's UC begins to stabilize under the administration of intravenous hydrocortisone. The acute gastrointestinal bleeding episodes subside, allowing for a transition to oral steroids. With the addition of aminosalicylates like mesalamine and immunomodulators such as azathioprine, the inflammation in his colon is brought under long-term control. Regular follow-up colonoscopies reveal gradual mucosal healing, and the frequency and severity of gastrointestinal bleeding decrease significantly.

Blood transfusions continue to effectively manage his thalassemia major, maintaining hemoglobin levels and preventing severe anemia. Iron chelation therapy, initiated to address iron overload from repeated transfusions, helps preserve liver and cardiac function. The patient follows a protocol for periodic transfusions and chelation therapy, with close monitoring to prevent transfusion-related complications.

In the long term, the patient benefits from a stable, multidisciplinary care plan for CS. Genetic counseling provides valuable insights and support to the patient and his family, helping them understand the condition and manage expectations. Thyroid hormone replacement therapy is regularly adjusted based on thyroid function tests, ensuring normal thyroid hormone levels and preventing symptoms of hypothyroidism.

Nutritional support and obesity management lead to gradual weight loss and improved overall health. Through dietary counseling and a tailored physical therapy regimen, the patient develops healthier eating habits and enhances his physical fitness. Psychological support and special education resources help address intellectual disability and developmental delays, improving his quality of life and providing effective coping strategies.

Regular monitoring identifies potential complications early, allowing for prompt intervention. The patient is closely observed for infections due to immunosuppressive therapy, cardiovascular health issues related to obesity and chronic illness, and liver dysfunction from iron overload. Periodic assessments ensure that any arising complications are managed effectively, maintaining his overall health.

With a structured follow-up schedule, the patient experiences fewer hospitalizations and better management of his chronic conditions. Weekly monitoring of hemoglobin levels and vital signs, monthly comprehensive blood work, and quarterly reviews of his treatment plan provide consistent and proactive care. Annual evaluations, including genetic counseling follow-up, bone density assessments, and full endocrinological and hematological reviews, offer comprehensive insights into his health, allowing for necessary adjustments to his care plan.

## Discussion

CS is a complex genetic disorder that affects multiple body systems. Specifically, this rare condition manifests with a spectrum of clinical features, including intellectual disability, characteristic facial appearance, and various physical abnormalities. Consequently, the differential diagnosis includes several other syndromic conditions due to their diverse manifestations. For example, Bardet-Biedl syndrome shares features such as obesity, retinal dystrophy, and developmental delays but can be distinguished by polydactyly and renal anomalies. Similarly, Prader-Willi syndrome is characterized by hypotonia, obesity, and developmental delays, with distinct features such as hyperphagia and specific facial dysmorphisms. Likewise, Angelman syndrome presents with intellectual disability and a happy demeanor, similar to CS, but includes ataxia and seizures. Additionally, Smith-Lemli-Opitz syndrome involves developmental delay, facial dysmorphisms, and growth retardation and is distinguishable by abnormalities in cholesterol metabolism. Furthermore, Alström syndrome features obesity and retinal dystrophy but differs from progressive hearing loss and cardiomyopathy [4-8].

UC needs to be differentiated from other inflammatory and non-inflammatory conditions of the gastrointestinal tract. Crohn’s disease can affect any part of the GI tract, presents with transmural inflammation, and commonly involves skip lesions. Infectious colitis is caused by bacterial, viral, or parasitic infections, usually identified through stool cultures. Irritable bowel syndrome is characterized by abdominal pain and altered bowel habits without the inflammatory markers seen in UC. Ischemic colitis typically occurs in older adults, is associated with sudden-onset abdominal pain, and is often linked to vascular diseases. Diverticulitis involves inflammation of the diverticula in the colon, presenting with localized pain, often in the lower left quadrant [9-13].

CS presents a variety of clinical features. Characteristic facial dysmorphisms include a high nasal bridge, a long philtrum, down-slanting palpebral fissures, and micrognathia (a small jaw). Motor and intellectual development are typically delayed. Central obesity often becomes noticeable during childhood. Progressive retinal dystrophy and myopia are common, leading to vision impairment. Intermittent neutropenia increases susceptibility to infections, and low muscle tone along with hypermobile joints are frequently observed [4,5,14].

The primary diagnosis of CS is going through clinical evaluation, with genetic testing used to identify mutations in the VPS13B gene. Specifically, the clinical diagnosis is based on characteristic facial features, developmental delays, retinal dystrophy, neutropenia, and truncal obesity. Identifying VPS13B mutations confirms the diagnosis. Consequently, the prognosis of CS varies depending on the severity of symptoms (Tables 2-3). While individuals may have a relatively normal lifespan, they often face challenges related to intellectual disability, visual impairment, and recurrent infections due to neutropenia. Therefore, early diagnosis and supportive care can significantly improve the quality of life and help manage complications effectively [15,16].

**Table 2:** Illustrates the diagnostic criteria of Cohen syndrome (clinical evaluation).

Diagnostic criteria of Cohen syndrome (clinical evaluation)	
History	Including family and genetic conditions
Physical exam	Focusing on: (1) Craniofacial characteristics, e.g., wave-shaped eyelids, short philtrum, thick hair, low hairline, thick eyebrows, high or narrow palate, microcephaly, and prominent upper central incisors; (2) growth and development abnormalities, e.g., speech and motor development delay; (3) ocular system, e.g., myopia, retinal dystrophy, and cataract; (4) gastrointestinal system, e.g., neonatal feeding difficulties; (5) nervous system, e.g., motor clumsiness, brisk reflexes, and cheerful disposition; (6) musculoskeletal system, e.g., hypotonia, slender limbs, and hypermobile joints.

**Table 3:** Illustrates the diagnostic criteria of Cohen syndrome (laboratory and other tests).

Diagnostic criteria of Cohen syndrome (laboratory and other tests)	
Laboratory tests	CBC, HbA1C, and lipid profile.
Ophthalmologic examination	Retinal examination. Visual acuity test.
Neurodevelopment assessment	Cognitive and behavior evaluation. Speech and language assessment.
Genetic testing	Including the VPS12B gene.

## Conclusions

We describe the complexity and multifaceted nature of Cohen syndrome in this case, emphasizing the need for accurate differential diagnosis and a comprehensive, multidisciplinary approach to management. The overlap of clinical features with other syndromic conditions underscores the importance of thorough clinical evaluation and genetic testing for an accurate diagnosis. Early recognition and targeted interventions, including the management of gastrointestinal complications and chronic conditions like thalassemia major, can significantly enhance patient outcomes. Continued follow-up and supportive care are crucial in improving the quality of life and managing the diverse manifestations of Cohen syndrome.
